# Agonist (P1) Antibody Converts Stem Cells into Migrating Beta-Like Cells in Pancreatic Islets

**DOI:** 10.4014/jmb.2209.09031

**Published:** 2022-10-28

**Authors:** Eun Ji Lee, Seung-Ho Baek, Chi Hun Song, Yong Hwan Choi, Kyung Ho Han

**Affiliations:** 1 Department of Biological Sciences and Biotechnology, Hannam University, Daejeon 34054, Republic of Korea; 2 Research Center for Bio-based Chemistry, Korea Research Institute of Chemical Technology (KRICT), Ulsan 44429, Republic of Korea

**Keywords:** Antibody, cell differentiation, periostin (POSTN), beta-like cells, insulin

## Abstract

Tissue regeneration is the ultimate treatment for many degenerative diseases, however, repair and regeneration of damaged organs or tissues remains a challenge. Previously, we showed that B1 Ab and H3 Ab induce stem cells to differentiate into microglia and brown adipocyte-like cells, while trafficking to the brain and heart, respectively. Here, we present data showing that another selected agonist antibody, P1 antibody, induces the migration of cells to the pancreatic islets and differentiates human stem cells into beta-like cells. Interestingly, our results suggest the purified P1 Ab induces beta-like cells from fresh, human CD34^+^ hematopoietic stem cells and mouse bone marrow. In addition, stem cells with P1 Ab bound to expressed periostin (POSTN), an extracellular matrix protein that regulates tissue remodeling, selectively migrate to mouse pancreatic islets. Thus, these results confirm that our in vivo selection system can be used to identify antibodies from our library which are capable of inducing stem cell differentiation and cell migration to select tissues for the purpose of regenerating and remodeling damaged organ systems.

## Introduction

Recently, the selection of gain-of-function agonists from intracellular combinatorial antibody libraries has emerged as a powerful method for the generation of antibodies that regulate cell fates. Given that a very large number of antibodies are tested (about 10^8^ different antibodies) in a single experiment, obtaining functional antibodies usually depends on development of a robust selection system. One very powerful selection system is autocrine based, where the target antigen and one member of the antibody library are expressed in the same cell, such that each cell becomes a selection system unto itself.

An important cell fate yet to be addressed by this methodology is cell migration, which is important in the development and immunological control of disease, including infection and autoimmunity, in the adult. The nature of each cell is decided by the adapted state of the cell where it resides. However, another level of efficiency could be achieved if cells were able to target both the chemistry and biology of a physiological task. For instance, an antibody could control cells and transport them to certain organs or tissues.

Self-renewal and multi-potency of bone marrow hematopoietic stem cells (HSCs) are essential abilities required for sustaining the human body. HSCs can replenish themselves as well as expand all lineages of blood cells throughout a lifetime. Further, hematopoietic progenitor cells (HPCs) migrate to site-specific microenvironments to repair tissue and restore vascular systems, and under some circumstances contribute to tumor formation. The interaction between HSCs and the microenvironment can initiate signal transduction, which leads to recruitment of site-specific cells [1]. When an antibody induces differentiation of HSCs to a specific cell fate, those cells can then migrate to specific organs as assigned by their programming.

In this study, we describe the autocrine-based selection of antibodies that induce bone marrow cells to enter a two-stage process where they first form beta-like cells that then migrate to the islet. To accomplish this, we used a novel selection system that capitalizes on the fact that, because of migration, the desired phenotype is self-selecting [2-4]. Usually, one needs to induce a signal that distinguishes some cells in a population from the bulk of the population. But, this strategy is still burdened by the problem that most of the cells in the population are uninteresting. By contrast, any cell that carries an antibody that induces it to migrate is at once enriched and interesting. We used an adoptive transfer protocol in these experiments. Bone marrow cells that were transformed with a 10^8^ combinatorial antibody library were transferred to irradiated mice, and those that migrated to the islet were recovered. Their antibody genes were harvested and the purified antibodies were shown to induce beta-like cells from fresh, human CD34^+^ cells and mouse bone marrow. The target of these antibodies was shown to be periostin (POSTN), which is of considerable interest because of this protein’s known role in organizing wound healing and mesenchymal cell proliferation [5, 6].

Given our increasing understanding of the role that beta-like cells play in metabolic diseases, such as diabetes, reagents that can increase their numbers in the islet may be therapeutically important.

## Materials and Methods

### Mouse Strains and Cell Lines

The C57BL/6J and B6.Cg-*Gt(ROSA)26Sor^tm9(CAG-ttdTomato)Hze^*/J mouse strains were purchased from Jackson Laboratories (USA). The 293T cell line was cultured in DMEM medium with 10% FCS and penicillin-streptomycin (Gibco-Invitrogen, USA). The Expi293F human cell line was cultured in the Expi293 Expression System (Gibco-Invitrogen). Human CD34^+^ hematopoietic stem cells (All-Cells) and mouse bone marrow cells were maintained in StemSpan Serum-Free Media with CC100 human cell culture cytokine cocktail (STEMCELL Technologies, Canada), and only serum-free media, or RPMI with 1% Fetal Bovine Serum. Mice were housed and handled according to protocols approved by the Institutional Animal Care and Use Committee at Hannam University (HNU2021-6).

### Combinatorial Antibody Library (ScFv Format) and Transduction

Single-chain Fv (ScFv) genes were derived from a naïve human combinatorial antibody library (1 × 10^11^ library diversity) and transferred into a lentiviral plasmid. The lentiviral antibody library was produced in the 293T cell line by co-transfection of lentiviral plasmids with the pCMVdR8.91 and pVSVg packaging plasmids at a 1:1:1 mass ratio. The mouse bone marrow cells were then incubated with the lentiviral antibody library for 3 days at 37°C.

### Mouse Bone Marrow Transplantation

Mouse bone marrow cells were transduced with the ScFv lentivirus library at a multiplicity of infection (MOI) of 2 and transplanted to lethally irradiated mice. The recipient mice with transplanted bone marrow were housed for 1–6 weeks. The pancreatic islets were perfused, fixed, harvested, and frozen at -80°C. The antibody DNA from the pancreatic islets was recovered by PCR with typical primer pairs customized for the lentiviral plasmid, and analyzed by electrophoresis.

### Purification of P1 Ab Proteins

The plasmid containing the ScFv-Fc affinity tag protein was transfected into human Expi293F cells for transient expression. P1 Ab proteins from the pooled supernatants were purified using HiTrap Protein G columns with an ÄKTAxpress Purifier FPLC System (GE, USA). The elution buffer was exchanged to phosphate-buffered saline (PBS) pH 7.4 and stored at 4°C.

### Western Blot

Cells were harvested with PBS and then lysed in cold RIPA lysis buffer. The cell lysates were centrifuged at 12,000 ×*g* for 15 min at 4°C. The proteins were denatured by boiling for 5 min at 95°C in SDS sample buffer, separated by SDS-polyacrylamide gel electrophoresis, and then transferred to nitrocellulose membranes using the Trans-Blot Turbo Transfer System (Bio-Rad, USA). For blocking, membranes were placed in tris-buffered saline with Tween 20 (TBST) containing 5% BSA for 30 min before being incubated with antibodies for 1 h. After washing the membranes several times with TBST, the blots were incubated with P1 Ab, POSTN protein (R&D Systems, USA), or secondary HRP-conjugated anti-β-actin antibody for 1 h. The membranes were then washed with TBST and developed with an enhanced chemiluminescence system. Phosphorylation was used with phospho-AKT and ERK (Cell Signaling Technology, USA).

### ELISA Assay

Human CD34^+^ hematopoietic stem cells were incubated with the selected P1 antibody for two weeks in vitro, prior to quantifying the level of insulin in the culture supernatant using the Human Insulin ELISA Kit (Invitrogen, USA) according to the manufacturer’s instructions.

### Flow Cytometry

Cells were incubated with anti-Insulin (R&D Systems), anti-PDX-1 (R&D Systems) and anti-Nkx-6.1 (DSHB, USA) before being washed and analyzed with an LSRII Flow Cytometer (Becton Dickinson, USA).

### Immunohistochemistry and Immune-Fluorescence Confocal Microscopy

Immunohistochemistry was analyzed on frozen pancreatic islet cells. Antibodies were used in 1x PBS with 4%horse serum and 0.2% Triton-X100. Guinea pig anti-Insulin (1:1000, Dako, USA), goat anti-PDX-1 (1:1000, R&D systems), mouse anti-Nkx-6.1 (1:1000, DSHB), or mouse anti-Glucagon-1 (1:1000, Thermo Fisher, USA) were used for analysis. Pancreatic islet cells were incubated overnight with each primary antibody, washed with PBS, and then incubated for 1 h with secondary antibody (1:250, Invitrogen). Pancreatic islet cells and coverslips were then mounted onto glass slides with anti-fade mounting medium containing DAPI (Thermo Fisher). Slides were then analyzed with a Zeiss LSM 710 laser scanning confocal microscope (Carl Zeiss, Germany).

### RNA Sequencing and Data Analysis

Total RNAs were purified from three replicates of isotype antibody-treated human CD34^+^ hematopoietic stem cells, human CD34^+^ hematopoietic stem cells treated with P1 Ab, and human CD34^+^ hematopoietic stem cells treated with vehicle Ab. Total RNA samples were converted into RNAseq libraries using the NEBNext Ultra Directional RNA Library Prep Kit for Illumina according to the manufacturer’s instructions. Statistical analyses were performed by edgeR (Bioconductor), and the differentially expressed genes were analyzed with false-discovery rates < 0.05, absolute fold change > 2 and averaged TPM (transcripts per million) > 1 in the two different conditions.

### Statistical Analysis

The data are expressed as the mean ± SD. Statistical significance was determined using a Student’s *t*-test. *p*-values of <0.05 were considered significant.

## Results

### In Vivo Selection of Antibodies that Migrate to Pancreatic Tissue

**Fig. 1:**
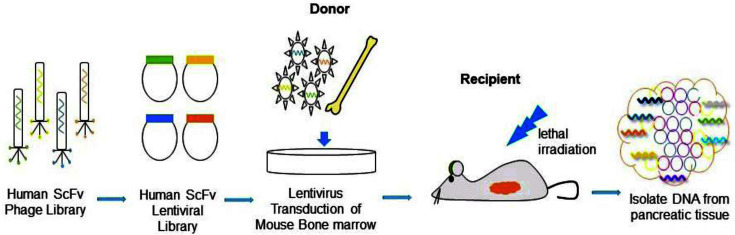
Scheme for the in vivo phenotype selection of the P1 antibody that induces differentiation and migration of mouse HSCs. Genes from a human ScFv phage library (10^8^ members) were cloned into a lentiviral vector to make a lentiviral membrane-tethered library in which antibody molecules are attached to the plasma membrane and displayed on the cell surface. Total mouse bone marrow cells were infected with the antibody library in vitro and transplanted into lethally irradiated C57BL/6J mice. The system is autocrine based because each cell has a different antibody and the putative target. After 7 days, the mouse pancreatic tissues were harvested and analyzed by PCR to identify any antibody genes in cells that traffic to the islet.

We previously designed an antibody selection system to select antibodies that manage cell fates using ScFv combinatorial libraries in vitro [1, 7, 8]. Additionally, a novel in vivo selection system was applied to isolate antibodies that induce stem cells to differentiate into microglia-like cells and adipocyte-like cells and selectively traffic to the brain and heart, respectively [2, 3]. We further investigated (Fig. 1) an unbiased in vivo selection system to isolate an agonist antibody that induces mouse bone marrow cells to migrate to the pancreatic tissue. To do this, an unbiased ScFv antibody library (membrane-tethered format) in lentiviruses containing about 108 distinctive combinations was used to infect total mouse bone marrow cells. The infected bone marrow cells were then transplanted into lethally irradiated mice to examine cell migration to the pancreatic islet. After 7 days, pancreatic tissues of mice perfused with PBS were harvested to extract genomic DNA. The human ScFv sequences that integrated into the cells were recovered by PCR. The P1 antibody gene was selected for further study.

### The Selected P1 Antibody Induces Migration of Cells to the Islet.

**Fig. 2:**
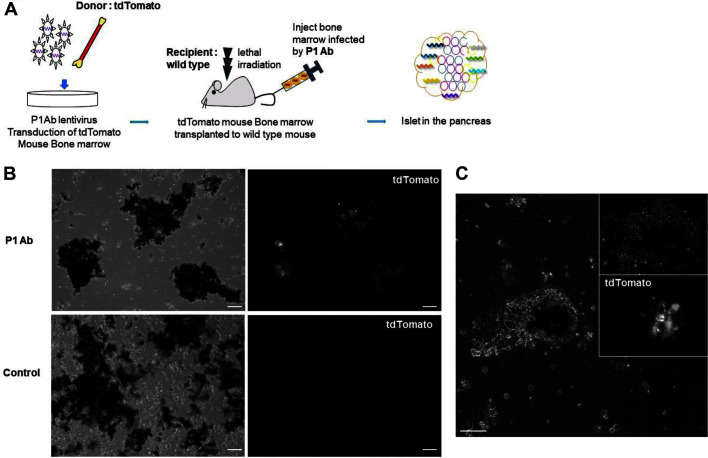
Antibody induced differentiation of HSCs into beta-like cells. (**A**) Scheme of the in vivo migration of cells from the bone marrow to the islet. tdTomato^+^ bone marrow cells were adoptively transplanted into lethally irradiated wildtype mice. P1 Ab (50 μg/mouse, i.p., two times/week) was injected into transplanted wild-type mice for 3 weeks. The mice were perfused, harvested, and analyzed by fluorescence microscope. (**B-C**) Significantly more tdTomato^+^ cells were identified by fluorescence microscope in the treated tissues as compared to controls (untreated tdTomato^+^ cells). These assays were repeated 3 times. The white box indicates the region magnified in the image to the right. Scale bars = 50 μm.

To show whether the P1 antibody could induce cell migration from the bone marrow to the islet, the P1 antibody gene was cloned into a mammalian expression plasmid and the P1 Ab was purified. Donor tdTomato^+^ bone marrow cells were adoptively transferred into irradiated C57BL6 mice. Then, P1 Ab (50 µg/mouse intraperitoneal injection (i.p.), two times/week) was injected into transplanted wild-type mice for 3 weeks. The pancreas was perfused, harvested, and analyzed for the presence of cells expressing tdTomato^+^ (Fig. 2A). Harvested islets were analyzed by fluorescence microscope. Microscope images showed donor tdTomato^+^ cells from mice injected with P1 Ab migrated to the islet (Figs. 2B and 2C).

### Purified P1 Antibody Differentiates Mouse Bone Marrow Stem Cells into Beta-Like Cells

**Fig. 3:**
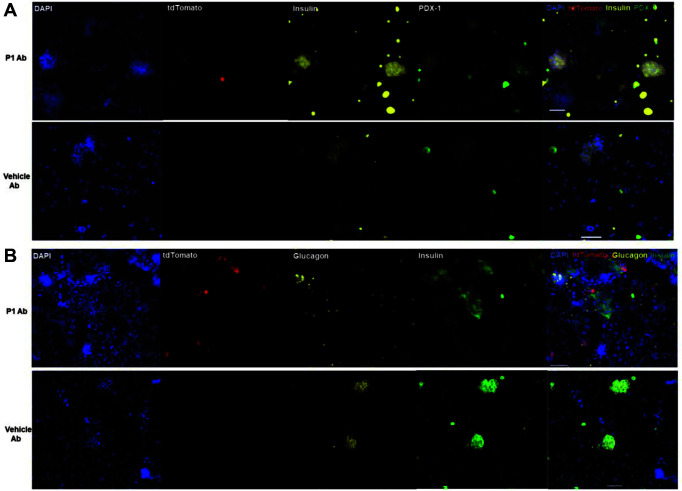
Antibody induced differentiation of bone marrow cells into beta-like cells. P1 Ab (50 μg/mouse, i.p., two times/week) was injected into lethally irradiated wild-type mice. After 3 weeks, the mice were perfused with PBS prior to harvesting the islets for immuno-fluorescence histochemistry. Islets were stained with DAPI, anti-Insulin, PDX-1 (beta cell marker), and glucagon (alpha cell marker). (**A**) Significantly more Insulin^+^ and PDX-1^+^ staining was identified in the P1 Abtreated dTomato^+^ tissues as compared to controls, suggesting that the beta-like cells migrated from the bone marrow to the islet. (**B**) Glucagon was not detected on the dTomato^+^ Insulin^+^ cells. Scale bars = 50 μm.

To verify that the P1 antibody genes induced bone marrow cells to traffic to the islet, we stained harvested islet cells with DAPI, anti-Insulin, and PDX-1. There were significantly more tdTomato^+^ Insulin^+^ PDX-1^+^ cells from mice injected with P1 Ab in the islets of irradiated mice as compared to vehicle antibodies (Fig. 3A). We next investigated whether migrated islet cells could be alpha cells. To this end, islet cells were stained with DAPI, anti-Insulin, and glucagon. However, glucagon, the alpha cell marker, was not detected on the tdTomato^+^ Insulin^+^ cells (Fig. 3B).

### The Selected P1 Antibody Differentiates Human Stem Cells into Insulin-Producing Beta-Like Cells

**Fig. 4:**
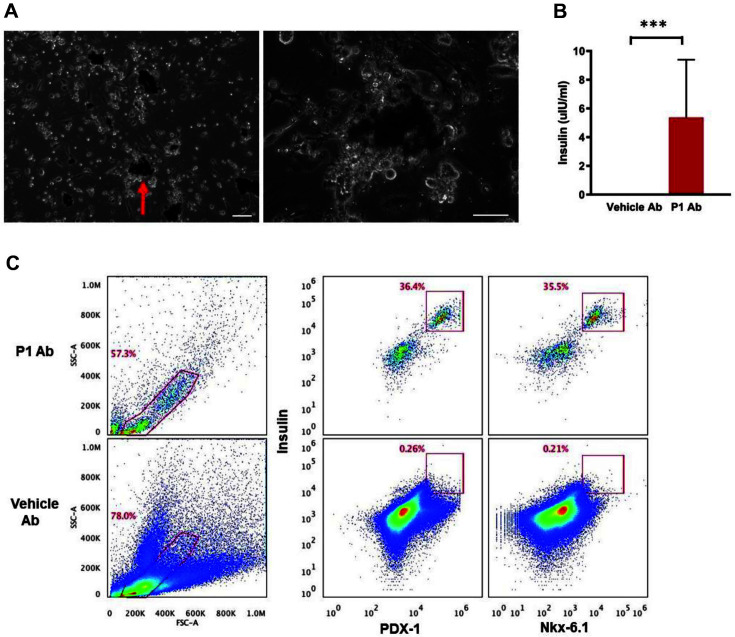
An agonist antibody induces beta-like cell differentiation. (**A**) Beta-like cells induced by the P1 Ab. Human CD34^+^ cells were incubated with P1 Ab (10 μg/ml) for 2 weeks. The red arrow in the image on the left indicates beta-like cells, which are magnified in the image to the right. (**B**) Insulin levels in the culture media were measured, and treatment with P1 Ab increased levels of insulin. . ***p-value, 0.0005; Student’s *t*-test. (**C**) Human CD34^+^ hematopoietic stem cells were incubated with P1 antibody and harvested islets were analyzed by flow cytometry. Cells were then stained with anti-Insulin, PDX-1, and Nkx-6.1. Scale bars = 50 μm.

To investigate if purified P1 antibody could transform cells, human CD34^+^ hematopoietic stem cells were incubated with the selected P1 antibody for two weeks in vitro. The purified P1 antibody induced human CD34^+^ hematopoietic stem cells to differentiate into cells with a cellular morphology resembling beta-like cells (Fig. 4A). To observe the in vitro effect of P1 on insulin production, we quantified insulin levels and found significantly more in P1 Ab-treated cells compared to vehicle antibody-treated cells (Fig. 4B).

The beta-like cells stained positive with beta cell marker antibodies, insulin, PDX-1, and Nkx-6.1 (Fig. 4C).

### Identification of a Novel Target

**Fig. 5:**
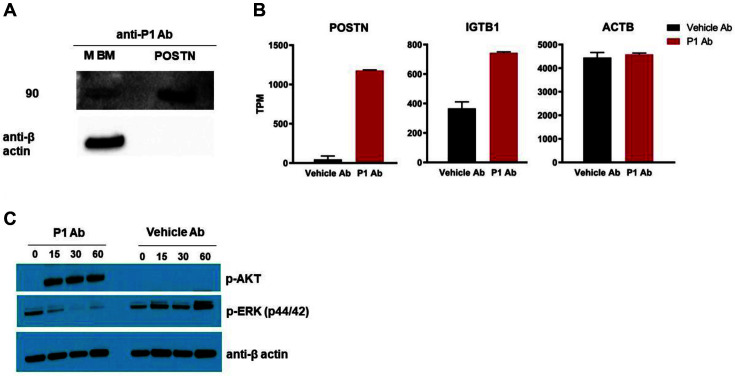
Identification of a novel antigen recognized by the P1 Ab. (**A**) The P1 Ab recognized human POSTN protein and murine bone marrow lysates when analyzed by western blotting. (**B**) Beta-like cell gene expression as determined by RNA sequencing analysis. Highly expressed beta cell markers from the RNAseq analysis (*n* = 3) are summarized as Transcripts Per Million mapped reads (TPM). (**C**) Human CD34^+^ cells were treated with P1 Ab or control isotype antibody, and cell lysates were assessed by western blotting using antibodies against phosphorylated (p-) AKT and ERK.

To determine the target protein captured by the P1 antibody, P1 antibody was produced in human Expi293F cells. Purified P1 antibody was mixed with mouse bone marrow, and P1 antibody-antigen complexes from cellular lysates were bound on a protein A/G column. Immune complex proteins that reacted with the antibody were recognized by silver staining of SDS-PAGE and immunoprecipitation mass spectrometry (MS). Six candidate proteins from peptide hits were selected above the background threshold. Periostin (POSTN) was one of the top hits from a homology search. POSTN was verified to be the target antigen of the P1 Ab by western blotting. The P1 Ab bound to purified POSTN protein, as well as mouse bone marrow, by western blotting (Fig. 5A). RNA transcripts of human CD34^+^ hematopoietic stem cells treated with purified P1 Ab were also analyzed and compared to the non-treated control cells in vitro. To determine transcripts that are expressed in beta-like cells, we compared our induced cells to expression data from previous reports [9]. Remarkably, we found genes in our induced cells that are highly expressed in beta-like cells, including IGTB1. These findings are consistent with data published by other groups (Fig. 5B). To determine whether binding of P1 Ab leads to the activation of signaling pathways, human CD34^+^ hematopoietic stem cells were treated with the P1 Ab, and cell lysates were assessed by western blotting with antibodies against non-phosphorylated and phosphorylated (p-) AKT and ERK. Consistent with their known role in beta cell differentiation, induction of p-AKT and suppression of p-ERK were observed in the cells stimulated with P1 Ab, but not with a vehicle antibody control (Fig. 5C).

## Discussion

In this study, we report on the selection of an agonist antibody that induces bone marrow cells to differentiate into beta-like cells. The identification of POSTN as the target antigen and a growing body of evidence suggest that POSTN, in addition to its more mechanical role as an extracellular matrix protein involved in tissue remodeling and bone regeneration, also plays a role in cell proliferation, stem cell activation, inflammatory reactions, and cellular signaling [10-15].

This surface localization is relevant to our studies using the lentivirus library to generate beta-like cells that migrate to the islet, because the antibodies are presented on the cell surface plasma membrane where they can bind to a membrane-related protein on a neighboring cell [7]. Furthermore, we have shown that extracellular soluble antibody added to stem cells induces the same differentiation program. Indeed, there is a certain parallelism with the role that periostin plays in signaling through integrin receptors, which promote cell adhesion, migration, and proliferation, and the putative migration of beta-like cells to sites of pancreatic injury [12, 15]. In both cases, periostin may be responsible for signal induction, possibly by generating unique chemotactic factors.

An issue raised by these studies concerns whether the bone marrow is a natural site for generation of beta-like cells. Beta cells derived from endoderm during embryonic and fetal development and pancreatic buds are induced, expanded, and differentiated into beta cells and other cells, such as alpha cells, delta cells, and duct cells [16-19]. Although beta cells can be derived from pluripotent stem cells, the key question that remains is, under what conditions do bone marrow-derived, beta-like cells migrate to the pancreatic islet in adult animals? It is now generally accepted that stem cells will migrate to sites of injury through stem cell homing [20, 21].

We don’t yet completely understand how an antibody agonist for periostin induces cell differentiation. Periostin is a marker of migrating cells, and in addition to its role in the mechanical aspects of cell migration, it is involved in promoting cell proliferation and differentiation [10, 22] as well as bone development and reconstruction by mesenchymal stem cells [22, 23]. It is also upregulated in allergies, inflammation, and cancer [11, 13]. For instance, periostin is required for the integrin-*β*1 receptor to suppress cell cycle arrest and apoptosis in in vivo and in vitro models [12]. The role of periostin in cell signaling is believed to be in the organization of signaling complexes inside the cell [24, 25].

Since periostin is on the cell surface as well, a similar organizing principle could explain our findings that soluble anti-periostin induces cell signaling in human CD34^+^ hematopoietic stem cells. In this case, the antibody could either stabilize or destabilize the complex leading to signal transduction. We are presently studying the nature of the members of the periostin complex on the cell surface of bone marrow cells.

Finally, in terms of migration and differentiation, a critical question remains about what comes first. Do cells migrate and then differentiate, or differentiate and then migrate? The mechanistic role of P1 Ab on periostin remains to be investigated in the future, separate study.

## References

[1] Han KH, Gonzalez-Quintial R, Peng Y, Baccala R, Theofilopoulos AN, Lerner RA (2016). An agonist antibody that blocks autoimmunity by inducing anti-inflammatory macrophages. FASEB J..

[2] Han KH, Arlian BM, Macauley MS, Paulson JC, Lerner RA (2018). Migration-based selections of antibodies that convert bone marrow into trafficking microglia-like cells that reduce brain amyloid beta. Proc. Natl. Acad. Sci. USA.

[3] Han KH, Arlian BM, Lin CW, Jin HY, Kang GH, Lee S (2020). Agonist antibody converts stem cells into migrating brown adipocyte-like cells in heart. Cells.

[4] Kang S, Park HW, Han KH (2022). Antibodies regulate dual-function enzyme IYD to induce functional synergy between metabolism and thermogenesis. Int. J. Mol. Sci..

[5] Walker JT, McLeod K, Kim S, Conway SJ, Hamilton DW (2016). Periostin as a multifunctional modulator of the wound healing response. Cell Tissue Res..

[6] Wu Z, Dai W, Wang P, Zhang X, Tang Y, Liu L (2018). Periostin promotes migration, proliferation, and differentiation of human periodontal ligament mesenchymal stem cells. Connect. Tissue Res..

[7] Xie J, Zhang H, Yea K, Lerner RA (2013). Autocrine signaling based selection of combinatorial antibodies that transdifferentiate human stem cells. Proc. Natl. Acad. Sci. USA.

[8] Zhang H, Yea K, Xie J, Ruiz D, Wilson IA, Lerner RA (2013). Selecting agonists from single cells infected with combinatorial antibody libraries. Chem. Biol..

[9] Diaferia GR, Jimenez-Caliani AJ, Ranjitkar P, Yang W, Hardiman G, Rhodes CJ (2013). β1 integrin is a crucial regulator of pancreatic beta-cell expansion. Development.

[10] Duchamp de Lageneste O, Colnot C (2019). Periostin in bone regeneration. Adv. Exp. Med. Biol..

[11] Izuhara K, Nunomura S, Nanri Y, Ono J, Takai M, Kawaguchi A (2019). Periostin: An emerging biomarker for allergic diseases. Allergy.

[12] Kormann R, Kavvadas P, Placier S, Vandermeersch S, Dorison A, Dussaule JC (2020). Periostin promotes cell proliferation and macrophage polarization to drive repair after AKI. J. Am. Soc. Nephrol..

[13] Sonnenberg-Riethmacher E, Miehe M, Riethmacher D (2021). Periostin in allergy and inflammation. Front. Immunol..

[14] Nie X, Shen C, Tan J, Wu Z, Wang W, Chen Y (2020). Periostin: A Potential therapeutic target for pulmonary hypertension?. Circ. Res..

[15] Wang Z, An J, Zhu D, Chen H, Lin A, Kang J (2022). Periostin: an emerging activator of multiple signaling pathways. J. Cell Commun. Signal..

[16] Borowiak M (2010). The new generation of beta-cells: replication, stem cell differentiation, and the role of small molecules. Rev. Diabet. Stud..

[17] Basile G, Kulkarni RN, Morgan NG (2019). How, When, and where do human beta-cells regenerate?. Curr. Diab Rep..

[18] Ji Z, Lu M, Xie H, Yuan H, Chen Q (2022). β cell regeneration and novel strategies for treatment of diabetes (Review). Biomed Rep..

[19] Kerper N, Ashe S, Hebrok M (2022). Pancreatic β-cell development and regeneration. Cold Spring Harb. Perspect. Biol..

[20] Liesveld JL, Sharma N, Aljitawi OS (2020). Stem cell homing: From physiology to therapeutics. Stem Cells.

[21] Yuan M, Hu X, Yao L, Jiang Y, Li L (2022). Mesenchymal stem cell homing to improve therapeutic efficacy in liver disease. Stem Cell Res. Ther..

[22] Zhu D, Zhou W, Wang Z, Wang Y, Liu M, Zhang G (2021). Periostin: an emerging molecule with a potential role in spinal degenerative diseases. Front. Med (Lausanne).

[23] Cobo T, Viloria CG, Solares L, Fontanil T, Gonzalez-Chamorro E, De Carlos F (2016). Role of periostin in adhesion and migration of bone remodeling cells. PLoS One.

[24] Rosselli-Murai LK, Almeida LO, Zagni C, Galindo-Moreno P, Padial-Molina M, Volk SL (2013). Periostin responds to mechanical stress and tension by activating the MTOR signaling pathway. PLoS One.

[25] Ouanouki A, Lamy S, Annabi B (2018). Periostin, a signal transduction intermediate in TGF-beta-induced EMT in U-87MG human glioblastoma cells, and its inhibition by anthocyanidins. Oncotarget.

